# Case Report: Family-mediated prognostic non-disclosure and psychological deterioration in terminal cancer

**DOI:** 10.3389/fpsyg.2026.1811856

**Published:** 2026-09-01

**Authors:** Pavithra Sankara Subramanian, Shrenik Ostwal

**Affiliations:** 1 Psychology Department, CMR University, Bangalore, India; 2 NMC Provita International Medical Center, Abu Dhabi, United Arab Emirates

**Keywords:** family dynamics, palliative care, prognostic non-disclosure, psychological distress, psycho-oncology, terminal cancer, case report, mental health

## Abstract

Prognostic non-disclosure is a common practice in Indian palliative care, often driven by family members' intentions to protect patients from emotional distress. However, the psychological consequences of sustained non-disclosure remain insufficiently documented within psycho-oncology literature. This case report, structured according to the CARE guidelines, describes the psychological trajectory of a 34-year-old male police officer with metastatic gastric carcinoma whose terminal prognosis was withheld at his family's request. Initially, the patient remained hopeful following a total gastrectomy and chemotherapy. However, upon readmission with progressive multi-level intestinal obstruction, hepatic dysfunction, and sepsis, he developed escalating anxiety and distress. The growing discrepancy between his lived bodily experience and the “white lies” provided by his family contributed to significant psychological deterioration, culminating in an episode of self-harm (intentional cut injury over the neck and hand) and terminal decline. This case illustrates how well-intentioned family-mediated non-disclosure may inadvertently intensify psychological suffering by undermining trust, autonomy, and emotional regulation. The report underscores the importance of culturally sensitive yet psychologically informed communication practices, early psychosocial assessment, and structured family counseling within palliative oncology care to preserve patient dignity and psychological stability at the end of life.

## Introduction

Cancer is not only a life-threatening medical condition but a profound psychological and existential stressor that affects and impacts patients across cognitive, emotional, behavioral, and relational domains (Wang and Feng, 2022). Within palliative care, psychological suffering often intensifies due to symptom burden, perceived loss of autonomy, and existential fear. The growing literature in psycho-oncology highlights that communication about diagnosis and prognosis plays a crucial role in shaping psychological adjustment (Walbaum et al., 2024). Within palliative care settings, psychological suffering often intensify due to symptom burden and increasing pain, and the perceived loss of autonomy, and concerns related to meaning, dignity, and death (Seiler et al., 2024).

The growing literature in psycho-oncology highlights that communication about diagnosis, prognosis, and treatment goals plays a crucial role in shaping patients psychological adjustment to cancer (Upadhyay et al., 2026). Prognostic awareness which is defined as a patient's understanding of the incurability or life-limiting nature of the illness has been associated with improved end-of-life decision-making, realistic goal setting, enhanced trust in healthcare providers, and in some cases the reduced psychological distress when communication is delivered sensitively (Arcadi et al., 2025). Conversely, lack of transparent communication may lead to mistrust, confusion, increased vulnerability to anxiety and depressive symptom and emotional dysregulation.

Regardless of the ethical emphasis on patient autonomy and informed consent in Western biomedical frameworks, prognostic non-disclosure remains a common practice in many non-western contexts including India (Glorioso, 2025). In these settings, families frequently act as primary decision-makers and may request that clinicians withhold prognostic information from patients, believing that disclosure could lead to emotional collapse, hopelessness, or hastened decline (Durosini and Pravettoni, 2023). These practices are often rooted in cultural values that prioritize collectivism, emotional protection, filial duty, and family cohesion over the individual autonomy (Knight et al., 2024).

Emerging evidence suggests that while family-mediated non-disclosure may be motivated by benevolent intentions, it can have unintended psychological consequences for patients, particularly when physical deterioration becomes increasingly apparent (Nääs et al., 2024). Individuals may perceive the discrepancies between their lived bodily experiences and the information provided to them, leading to heightened anxiety, suspicion, agitation, and emotional distress (Gentsch and Kuehn, 2022). Meanwhile in the advanced illnesses, this incongruence between reality and communicated information can contribute to existential distress, identity disruption, feelings of hopelessness and in extreme cases, suicidal ideation or behavioral dysregulation may occur (Collett Wright, 2025).

In Indian palliative care settings, clinicians often face complex ethical dilemmas in balancing the respect for cultural norms, family wishes, and the psychological wellbeing of the patient (Veena et al., 2025). Within the limited mental health literacy, stigma surrounding the psychological symptoms, and fear of emotional breakdown further complicates the timely psychosocial assessments and interventions (Choudhary et al., 2024). Nevertheless, there remains a relative paucity of detailed clinical case reports that explicitly documents the psychological trajectory of patients subjected to prolonged prognostic non-disclosure, particularly in younger adults with strong occupational and identity-based self-concepts (Sutar and Chaudhary, 2022).

The current case report aims to address this gap by illustrating the psychological deterioration of a young adult male with metastatic gastric carcinoma whose prognosis was withheld as per the family's request. Through a detailed psychosocial and clinical narrative, this report examines how sustained non-disclosure interacted with progressive physical decline to produce escalating anxiety, mistrust, agitation, fear, identity conflict, and suicidal behavior. By situating this case within the broader psycho-oncology and cultural ethics literature, the report aims to highlight the importance of culturally sensitive yet psychologically informed communication strategies, early psychosocial intervention, and structured family counseling in palliative oncology care.

## Patient information

The patient was a 34-year-old married male police officer with advanced metastatic gastric carcinoma (UICC Stage IV), status post total gastrectomy and chemotherapy, who was admitted with disease progression complicated by multi-level intestinal obstruction, hepatic dysfunction, and sepsis. His relevant past medical interventions included a total gastrectomy followed by a course of chemotherapy.

He had no documented prior history of psychiatric illness, substance use disorder, or self-harm. His primary support system consisted of his young wife, parents, and elder brothers. Due to historical extended family strains, the patient exhibited deep, exclusive trust in his wife for emotional stability and medical translation. Following his initial surgical and chemotherapeutic interventions, he expressed strong hopes of restoring his lifestyle and returning to active police duty.

Prior to disease progression, the patient demonstrated predominantly adaptive coping strategies. He remained optimistic regarding treatment, expressed a strong desire to return to active police service, and derived psychological strength from his occupational identity and close relationship with his wife. His primary coping resources included hope for recovery, emotional reliance on his spouse, and confidence in the treating medical team. As his illness progressed and the discrepancy between his physical deterioration and the information provided by family members widened, these coping mechanisms gradually became overwhelmed, contributing to increasing emotional distress, mistrust, and behavioral dysregulation.

## Timeline

During his initial oncological treatment, the patient was functionally independent, coherent, and maintained adaptive coping strategies. However, his condition severely deteriorated, leading to a critical readmission. Clinical examination and diagnostic imaging during this readmission revealed advanced cancer progression complicated by multi-level intestinal obstruction, hepatic dysfunction, and sepsis.

As his physical discomfort intensified, psychological distress manifested visibly. The patient became intensely restless and highly dependent on his wife's physical presence. This psychological and physical decline culminated when the patient presented with a self-intentional cut injury over his neck and hand, necessitating an immediate shift to the Medical Intensive Care Unit (MICU). Upon terminal decline, physical findings included aspiration pneumonia, worsening shock, and refractory acidosis.

The following table correlates the patient's somatic progression with the family's information management and his resulting psychological trajectory, in accordance with CARE guidelines. The patient's clinical course, family-directed information management, and corresponding psychological trajectory are summarized in Table 1.

**Table 1:** Timeline of clinical progression, family-mediated information management, and psychological trajectory of a patient with metastatic gastric carcinoma experiencing prognostic non-disclosure.

Date/Phase	Somatic progression and interventions	Information management and family directives	Psychological trajectory and behavioral manifestations
Initial phase	Diagnosis of Ca stomach with metastasis. Interventions: total gastrectomy and chemotherapy.	Full disclosure of initial diagnosis; active curative-intent treatment.	Patient maintains optimism and adaptive coping. Expresses strong desire to return to work. Deep trust in the medical team is established.
Readmission	Escalating abdominal pain, severe weakness. Scans reveal multi-level intestinal obstruction and hepatic dysfunction.	Terminal prognosis delivered to wife and brother. Family rigidly mandates non-disclosure to the patient.	Wife frames the hospital admission as a “regular check-up” and later as routine “pain management.” Patient relies heavily on wife's narrative.
Symptomatic Escalation	Sepsis develops; severe peripheral edema and ascites manifest.	Wife explains physical swelling is “because of medications.” When pressed by the patient, she claims, “I do not know, the doctor won't tell me too.”	Extreme physical weakness and pain. Internal conflict arises between his bodily decay and the provided narrative. Trust begins to fracture.
Acute Crisis	Patient shifted to MICU following a self-intentional cut injury over his neck and hand.	Relatives are explicitly counseled on the deteriorating condition (aspiration pneumonia, sepsis). Family opts for comfort care, refusing intubation/resuscitation.	Acute distress culminating in self-harm. Manifestation of severe helplessness, existential despair, and loss of systemic control.
Terminal Decline	Progressive shock and refractory acidosis. Patient expires.	Prioritization of extreme symptom relief and emotional containment.	Formal psychiatry opinion obtained, noting a depressive disorder. Terminal multi-organ dysfunction leading to death.

## Diagnostic assessment

The somatic diagnosis of advanced metastatic gastric carcinoma complicated by multi-level intestinal obstruction and hepatic dysfunction was established via imaging, clinical chemistry, and surgical history.

Psychological assessments were conducted continuously by an interdisciplinary team. Diagnostic methods utilized included the Mini-Mental State Examination (MMSE), Mental Status Examination (MSE), detailed case history, clinical interviews, and daily behavioral observations. The primary diagnostic challenge was differentiating psychological distress induced by non-disclosure from acute organic delirium secondary to his hepatic dysfunction and sepsis. Ultimately, a formal psychiatry consultation on his final day noted a severe depressive disorder. The episode of self-harm was interpreted clinically as an acute behavioral manifestation of profound helplessness, unresolved physical pain, and systemic mistrust resulting from informational deprivation.

## Therapeutic interventions

Somatic management in the palliative phase, particularly after his shift to the MICU focused strictly on comfort care as opted by the family (who declined intubation and resuscitation). He was managed conservatively with antibiotics, analgesics, hepatoprotective measures, and feeding via total parenteral nutrition (TPN).

Psychosocial interventions were delivered by an interdisciplinary team comprising a clinical psychologist, palliative care nurses, and the primary pain and palliative care physician. The team utilized behavioral techniques, clinical interviews, and supportive counseling. From the time of readmission until the patient's death, the clinical psychologist conducted daily bedside counseling sessions with the patient's wife and brother. These sessions focused on psychoeducation regarding the patient's psychological distress, emotional support for family members, discussion of the ethical implications of prognostic non-disclosure, preparation for end-of-life care, and repeated encouragement to consider a gradual, culturally sensitive disclosure of the patient's prognosis. Despite these repeated interventions, the family remained consistent in their decision to withhold prognostic information.

The clinical psychologist focused on supportive psychotherapy aimed at reducing emotional distress, facilitating emotional expression, providing reassurance, reality-oriented communication appropriate to the patient's level of awareness, and strengthening coping with progressive physical decline. Family sessions primarily involved psychoeducation regarding psychological distress, anticipatory grief, communication strategies, and the potential psychological consequences of sustained prognostic non-disclosure. Crisis intervention was provided following the self-harm episode in collaboration with the psychiatry and palliative care teams.

## Follow-up and outcomes

The patient succumbed to multi-organ dysfunction secondary to advanced carcinoma of the stomach.

Recognizing the severe risk of complicated grief associated with prolonged deception, the primary oncologist and clinical psychologist conducted targeted bereavement follow-up counseling. The team met separately with the young wife and the broader family (including the brother). The wife was encouraged to openly express her suppressed grief, seek ongoing therapy, and integrate back into the workforce to process her trauma. The brother and extended family were explicitly counseled on their responsibility to support the wife through her bereavement. Given her young age, the team also initiated discussions with the family regarding the importance of supporting her eventual reintegration into a normal life, including the possibility of future remarriage.

During bereavement follow-up, the patient's wife expressed profound emotional distress and difficulty processing the rapid deterioration and death of her husband. Family members acknowledged the emotional burden associated with maintaining prognostic non-disclosure and participated in discussions regarding grief, future psychosocial support, and family responsibilities. Although formal long-term psychological follow-up was not available, the family demonstrated willingness to engage with bereavement counseling and supportive services.

## Discussion

This case intimately details the catastrophic psychological cascade triggered by prognostic non-disclosure. It specifically highlights the friction between the patient's occupational identity, the mechanics of familial deception, the moral distress of the healthcare team, and the downstream impacts on bereavement.

The “White Lies” and the Investigative Paradox:

A central narrative paradox in this case is why a trained police officer a profession defined by an investigative mindset and a demand for truth failed to uncover his own terminal diagnosis. This can be attributed to two factors: severe physical debilitation and profound interpersonal trust. Because the patient had successfully navigated his initial gastrectomy and chemotherapy under the care of this specific medical team, a deep, foundational rapport had been established. His extreme physical weakness and severe pain left him entirely reliant on this pre-existing trust.

To maintain the non-disclosure directive, his wife was forced to construct a continuous series of “white lies.” She initially framed his readmission as a “regular check-up” and later as routine “pain management.” When severe edema developed, she explained it away as “because of medications.” When the patient's investigative instincts flared, resulting in direct questioning, she utilized a strategy of feigned systemic ignorance, claiming, “I do not know, the doctor won't tell me too.” This elaborate fiction ultimately crumbled as his organ systems failed, leading to severe behavioral dysregulation and the acute self-harm incident over his neck and hand as his last available mechanism to exert control.

### Family-centered shared decision making (FCSDM)

In Asian and collectivist medical paradigms, Family-Centered Shared Decision Making (FCSDM) often replaces Western individual autonomy. Under this framework, the family assumes the emotional burden of terminal knowledge as an act of filial duty and protection. However, this case demonstrates the catastrophic failure of FCSDM when it demands complete passivity from a patient whose core identity is built on authority and control. The rigid application of FCSDM severed the authentic clinical partnership, leaving the patient isolated with his decaying body.

### Staff moral distress

The rigid enforcement of the family's non-disclosure directive generated significant moral distress among the palliative care and nursing staff. The nurses were forced to absorb the patient's escalating frustration, strictly forbidden from defending their care by revealing the underlying biological reality. This forced complicity in deception creates an ethically toxic environment that directly undermines the therapeutic nurse-patient relationship.

### Bereavement and prolonged grief

Finally, this case underscores how non-disclosure negatively impacts the quality of dying, which subsequently complicates caregiver bereavement. By maintaining a facade of optimism, the wife was entirely robbed of the opportunity for anticipatory grieving, shared goodbyes, and mutual closure. The post-mortem bereavement interventions executed by the clinical psychologist and oncologist were critical in mitigating this trauma, emphasizing the necessity of viewing the family unit as the ultimate patient in palliative oncology.

## Limitations

This report describes a single retrospective clinical case and therefore the findings cannot be generalized to all patients experiencing prognostic non-disclosure. The patient's rapidly deteriorating medical condition and fluctuating cognitive status limited the opportunity to obtain a comprehensive first-person narrative and repeated standardized psychological assessments throughout the illness trajectory. In addition, some clinical observations were derived from interdisciplinary documentation and family interactions rather than formal psychometric measures. Although the case illustrates important psychological and ethical issues surrounding family-mediated prognostic non-disclosure, further prospective studies and larger case series across diverse cultural settings are required to better understand the psychological consequences and to inform evidence-based communication practices in palliative oncology.

## Patient perspective

In the beginning, a direct patient narrative could not be obtained due to the patient's rapid deterioration and incomplete awareness of his prognosis. Throughout the course of his illness, his verbal expressions reflected confusion, fear and a desire of transparency. His at multiple stages, he requested explanations regarding his worsening health condition and repeatedly expressed a desire to return home. He became agitated, became verbally confrontational with nursing staff, demonstrated reduced adherence to treatment recommendations as a way to show his helplessness and lack of control and vulnerability. The episode of self-harm represented the culmination of his psychological distress and reflected his desperate need for emotional reassurance and understanding.

## Clinical findings

In the initial phases of treatment, the patient presented as physically stable, with ability to walk independently, eat without assistance, and engage in daily activities. His speech was coherent and he maintained a positive demeanor. He demonstrated no overt psychiatric symptoms and displayed adaptive coping strategies. However, over time he began experiencing abdominal pain, dyspnoea, swelling of legs and abdomen, and difficulty swallowing. The symptoms significantly impaired his mobility and ability to maintain normal routine. As physical discomfort intensified, the psychological distress became increasingly evident. He became increasingly anxious, restless, and preoccupied with the causes of his worsening condition. He repeatedly questioned the reasons for his worsening condition and asked whether the investigations and medical tests had revealed any serious findings. In keeping with the family's request for prognostic non-disclosure, these concerns were answered with reassuring explanations that attributed his symptoms to treatment-related complications and routine medical management rather than disease progression.

As the prognosis intensified, he exhibited agitation, irritability, aggressive verbal interactions with nursing staff, withdrawal, fluctuating orientation, paranoid ideas about medical neglect, and eventually hallucinations and suicidal ideation.

As his illness advanced, additional behavioral and cognitive changes were documented during routine clinical observations. The patient increasingly exhibited reduced initiation of communication, often responding briefly or with delayed answers, and required repeated prompts during interactions. Periods of marked psychomotor slowing alternated with episodes of heightened agitation, during which he would attempt to remove medical devices or insist on leaving the ward. His ability to sustain attention during conversations and care procedures diminished, and he appeared easily distracted by environmental noise or movement. Staff noted increased dependency on familiar caregivers, particularly his wife, and heightened distress when separated from her presence, manifesting as restlessness, verbal irritability, and repeated calls for reassurance.

In the later stages, the patient showed fluctuating awareness of his surroundings, with occasional misinterpretation of staff intentions and difficulty recognizing the purpose of ongoing medical interventions. He demonstrated reduced frustration tolerance, becoming verbally reactive during routine nursing care such as repositioning, hygiene assistance, or medication administration. Episodes of confusion were more pronounced during evenings and nights, with transient disorientation and perceptual disturbances noted on multiple occasions. His overall engagement with care progressively declined, necessitating closer supervision to ensure comfort, safety, and adherence to basic supportive measures.

## Significance

Despite increasing attention to psychological distress in advanced cancer, the specific mental health consequences of prolonged prognostic non-disclosure remain insufficiently examined within psycho-oncology literature, particularly in non-Western and collectivist cultural contexts. Existing research has primarily focused on the benefits and challenges of truth-telling, prognostic awareness, and advance care planning, often emphasizing decision-making outcomes rather than the evolving psychological experiences of patients who remain unaware of their prognosis (Allison et al., 2023). This case report contributes to the literature by providing a detailed, clinically grounded account of the psychological trajectory of a terminally ill patient subjected to sustained family-mediated non-disclosure.

The present case is significant in documenting the temporal progression of psychological deterioration, capturing changes in emotional regulation, cognitive functioning, behavioral responses, and existential distress as the illness advanced. Prior studies have demonstrated that uncertainty and lack of illness understanding are associated with increased anxiety, depression, and demoralization in patients with advanced cancer (Hao et al., 2023). However, such findings are often derived from cross-sectional designs. By contrast, this report offers longitudinal clinical insight into how discrepancies between patients' lived bodily experiences and communicated information may undermine trust, emotional containment, and psychological stability over time.

This case further contributes by situating psychological distress within a culturally embedded framework of family-centered decision-making. In many Indian and Asian healthcare contexts, families commonly request non-disclosure as a protective strategy rooted in collectivist values and concerns about emotional harm (Lau, 2023). While culturally sensitive care is essential, this case illustrates how rigid adherence to non-disclosure may inadvertently limit opportunities for psychological preparation, meaning-making, and preservation of dignity at the end of life (Veena et al., 2025). The findings support emerging calls for negotiated or gradual disclosure approaches that balance cultural values with patient psychological wellbeing.

Finally, the case has important clinical implications for psycho-oncology and palliative care practice. It highlights under-recognized risk factors for severe psychological distress, including sustained prognostic uncertainty, identity disruption in younger patients with strong occupational self-concepts, and restricted access to psychosocial interventions due to family resistance (Kirkbride et al., 2024). Prior literature indicates that unresolved distress, perceived loss of autonomy, and lack of emotional support are associated with increased vulnerability to suicidal ideation in advanced cancer populations (Huda et al., 2022). By explicitly documenting these dynamics, this report underscores the importance of early psychosocial assessment, interdisciplinary collaboration, and proactive family counseling to mitigate preventable psychological suffering in terminal illness.

## Conclusion

The case illustrates the impact of prognostic non-disclosure in terminal cancer and the profound psychological impact and emphasis on the importance of transparent, culturally sensitive communication practices. The patient's psychological deterioration, including anxiety, mistrust, agitation, frustration, despair, identity conflict, and self-harm episode, occurred within the sphere of inconsistent information, family-mediated protection and escalating physical decline. The report presents the need for early psychosocial assessment, structured communication frameworks, family counseling and collaborative ethical deliberation in palliative and cancer care. Addressing emotional and cultural dynamics alongside medical needs, clinicians can help patients maintain dignity, trust and emotional stability at the end of life.

## Data Availability

The datasets presented in this article are not readily available because of ethical and privacy restrictions. Requests to access the datasets should be directed to the corresponding author.
